# Early mobilization versus plaster immobilization of simple elbow dislocations: a cost analysis of the FuncSiE multicenter randomized clinical trial

**DOI:** 10.1007/s00402-019-03309-1

**Published:** 2019-11-23

**Authors:** Esther M. M. Van Lieshout, Gijs I. T. Iordens, Suzanne Polinder, Denise Eygendaal, Michael H. J. Verhofstad, Niels W. L. Schep, Dennis Den Hartog, FuncSiE Trial Investigators, Roelf S. Breederveld, Maarten W. G. A. Bronkhorst, Jeroen De Haan, Mark R. De Vries, Boudewijn J. Dwars, Robert Haverlag, Sven A. G. Meylaerts, Jan-Willem R. Mulder, Peter Patka, Kees Jan Ponsen, W. Herbert Roerdink, Gert R. Roukema, Inger B. Schipper, Michel A. Schouten, Jan Bernard Sintenie, Senail Sivro, Wim E. Tuinebreijer, Johan G. H. Van den Brand, Frits M. Van der Linden, Hub G. W. M. Van der Meulen, Egbert J. M. M. Verleisdonk, Jos P. A. M. Vroemen, Marco Waleboer, W. Jaap Willems

**Affiliations:** 1 Trauma Research Unit, Department of Surgery, Erasmus MC, University Medical Center Rotterdam, P.O. Box 2040, 3000 CA Rotterdam, The Netherlands; 2 Department of Public Health, Erasmus MC, University Medical Center Rotterdam, P.O. Box 2040, 3000 CA Rotterdam, The Netherlands; 3 Upper Limb Unit, Department of Orthopaedic Surgery, Amphia Hospital, P.O. Box 90158, 4800 RK Breda, The Netherlands; 4 Trauma Unit, Department of Surgery, Academic Medical Center, P.O. Box 22660, 1100 DD Amsterdam, The Netherlands

**Keywords:** Cost-effectiveness, Cost utility, Elbow dislocation, Function, Quality of life

## Abstract

**Introduction:**

The primary aim was to assess and compare the total costs (direct health care costs and indirect costs due to loss of production) after early mobilization versus plaster immobilization in patients with a simple elbow dislocation. It was hypothesized that early mobilization would not lead to higher direct and indirect costs.

**Materials and methods:**

This study used data of a multicenter randomized clinical trial (FuncSiE trial). From August 25, 2009 until September 18, 2012, 100 adult patients with a simple elbow dislocation were recruited and randomized to early mobilization (immediate motion exercises; *n* = 48) or 3 weeks plaster immobilization (*n* = 52). Patients completed questionnaires on health-related quality of life [EuroQoL-5D (EQ-5D) and Short Form-36 (SF-36 PCS and SF-36 MCS)], health care use, and work absence. Follow-up was 1 year. Primary outcome were the total costs at 1 year. Analysis was by intention to treat.

**Results:**

There were no significant differences in EQ-5D, SF-36 PCS, and SF-36 MCS between the two groups. Mean total costs per patient were €3624 in the early mobilization group versus €7072 in the plaster group (*p* = 0.094). Shorter work absenteeism in the early mobilization group (10 versus 18 days; *p* = 0.027) did not lead to significantly lower costs for loss of productivity (€1719 in the early mobilization group versus €4589; *p* = 0.120).

**Conclusion:**

From a clinical and a socio-economic point of view, early mobilization should be the treatment of choice for a simple elbow dislocation. Plaster immobilization has inferior results at almost double the cost.

## Introduction

The elbow is the second most commonly dislocated joint in adults and mostly occurs in young and active persons, thus affecting the working population [1–3]. A simple elbow dislocation (no associated fractures) is a disabling injury which causes considerable pain and loss of range of motion in the short term, which impedes the ability to perform daily activities such as work [4].

Previous studies suggested that early mobilization may give superior functional results [5–11]. The FuncSiE trial compared clinical outcome of early mobilization and plaster immobilization in patients with a simple elbow dislocation. The results of this study showed that early mobilization resulted in earlier recovery of elbow function and work resumption [12].

These results justify the design of a treatment guideline advocating early mobilization from a clinical point of view. However, there are no high-quality studies that report the burden of simple elbow dislocations on direct and indirect health care costs, let alone to what extent early mobilization would be able to reduce these costs. We performed a cost analysis of the FuncSiE randomized controlled trial to assess the direct and indirect costs and the cost-effectiveness of early mobilization versus plaster immobilization in patients with a simple elbow dislocation. It was hypothesized that early mobilization would not lead to higher costs.

## Materials and methods

### Settings and participants

This cost analysis used data of a multicenter randomized clinical trial comparing early mobilization with plaster immobilization in patients after a simple elbow dislocation (FuncSiE trial). The trial is registered at the Netherlands Trial Register (NTR2025). The results of this study and the study protocol can be read elsewhere [12, 13]. The study was approved by the Medical Research Ethics Committee. All patients gave written informed consent.

Adult patients (aged 18 years or older) with a simple elbow dislocation were recruited from August 25, 2009 until September 18, 2012. Polytraumatized patients, patients with recurrent or open dislocation, additional traumatic injuries of the affected arm, an indication for surgical intervention, impaired elbow function pre-trauma, previous surgery or fractures involving the elbow, or expected problems with maintaining follow-up were excluded.

### Randomization and masking

Patients were randomly assigned to receive early mobilization (early active movements within the limits of pain, started immediately after closed reduction as tolerated) or plaster immobilization (immobilization in a long arm cast for 3 weeks followed by movements within the limits of pain). In both groups, mobilization was supervised by a physical therapist following a guideline that was designed for this study. Further details concerning the randomization procedure and both interventions can be read in the original article and the study protocol [12, 13].

### Assessments and follow-up

Data were obtained during out-patient visits at 1, 3 and 6 weeks, and at 3, 6, and 12 months after randomization. During these visits, patients completed questionnaires concerning health-related quality of life and a health care consumption questionnaire. This questionnaire included questions on the number of visits to the physical therapist, general practitioner, and medical specialist, admission to hospital, rehabilitation center or nursing home, medication use, and the use of home care. The questionnaire also included questions concerning work absenteeism and resumption. Questionnaire data were supplemented with data from the patients’ medical files.

The primary outcome measure for this analysis was total costs, consisting of direct costs (i.e., costs for treatment and intramural care) and indirect costs (i.e., costs for lost production). Secondary outcome measures included the health-related quality of life using the EuroQol-5D (EQ-5D) [14] and Short Form-36 (SF-36), which are both validated [15]. The use of the EQ-5D is recommended for assessing quality of life in trauma patients especially for economic assessments [16, 17]. The scores for the physical and mental components of the SF-36 were converted to a norm-based score and compared with the norms for the general population of the United States [15]. As there were no significant differences in quality of life scores between the two groups at 1 year, no cost-effectiveness and cost–utility ratio could be calculated. Therefore, a cost-minimization analysis was performed.

### Cost measurement

The total direct and indirect costs of both treatments were analyzed from a societal perspective and included: (1) in-hospital care costs which were subdivided into costs for the primary intervention, costs during follow-up, and costs for diagnosis and treatment of adverse events; (2) out of hospital care costs for rehabilitation; and (3) indirect costs due to productivity loss. Costs were calculated by multiplying the volumes with the corresponding unit prices (Table 1). Hospital costs for the primary intervention and costs during follow-up consisted of fixed and variable costs. As no patients were admitted to a nursing home or rehabilitation clinic, these costs were zero for all patients. Lost productivity was represented by the hours of work absence.

**Table 1:** Data sources, sources of valuation and unit prices of all cost categories

Cost categories	Unit	Number of fixed units for EM/PI	Source of data	Source of valuation	Unit price (€)
*Hospital costs—primary intervention*					
Emergency department visit	Visit	1/1	Hospital registry	Cost manual	€161.12
Radiology/diagnostics					
X-ray^a^	X-ray	3/3	Hospital registry	NZa	€51.07
CT scan	CT scan	Variable	Hospital registry	NZa	€202.14
MRI	MRI	Variable	Hospital registry	NZa	€256.79
Ultrasound	Ultrasound	Variable	Hospital registry	NZa	€76.64
Arthrogram	Arthrogram	Variable	Hospital registry	NZa	€126.86
Anesthetics/sedation	Case	Variable	Study/hospital registry	CVZ	€2.00^b^
Plexus block/regional anesthesia	Case	Variable	Study/hospital registry	Hospital data	€82.00
Reduction in operating room					
Surgeon	Minute	Variable	Study/hospital registry	Cost manual	€2.41^c^/€1.83^d^
Anesthesiologist	Minute	Variable	Hospital registry	Cost manual	€2.41^c^/€1.83^d^
Operating room^e^	Minute	Variable	Study/hospital registry	Hospital data	€14.75^c^/€11.87^d^
Treatment					
Plaster	Plaster	0/1	Study registry	Hospital data	€127.18
Plaster change at 1 week	Plaster	0/1	Study/hospital registry	Hospital data	€158.60
Pressure bandage	Pressure bandage	1/0	Study registry	Hospital data and https://www.medischservice.nl	€15.21
Sling	Sling	1/1	Study registry	Hospital data and https://www.medischservice.nl	€15.00
Admission days	Days	Variable	Study/hospital registry	Cost manual	€464.15^c^/€613.53^d^
Visit out-patient clinic or plaster room^f^	Visit	2/2	Study/hospital registry	Cost manual	€68.29^c^/€137.64^d^
*Hospital costs—follow-up*					
Visit out-patient clinic^g^	Visit	4/4	Study/hospital registry	Cost manual	As displayed above
Radiology/diagnostics^h^	Study	1/1	Study/hospital registry	NZa	As displayed above
*Hospital costs—adverse events/revision surgery*					
Visit out-patient clinic or plaster room^i^	Visit	Variable	Study/hospital registry	Cost manual	As displayed above
Radiology/diagnostics^j^	Study	Variable	Study/hospital registry	NZa	As displayed above
Operating room^e^	Minutes	Variable	Hospital registry	Hospital data	As displayed above
Surgeon	Minutes	Variable	Hospital registry	Cost manual	As displayed above
Anesthesiologist	Minutes	Variable	Hospital registry	Cost manual	As displayed above
Type of surgery					
Arthrolysis^e^	Procedure	Variable	Hospital data	Hospital data	€47.10
Ulnar nerve release^e^	Procedure	Variable	Hospital data	Hospital data	€56.91
Arthroscopy wrist^e^	Procedure	Variable	Hospital data	Hospital data	€220.17
Admission days	Days	Variable	Study/hospital registry	Cost manual	As displayed above
*Out of hospital costs—follow-up/rehabilitation*					
General practitioner	Visits	Variable	Study registry	Cost manual	€29.88
Physical therapy	Visits	Variable	Study registry	Cost manual	€38.41
Home care	Hours	Variable	Study registry	Cost manual	€37.35
*Indirect cost*					
Work absenteeism males < 35 years	Hours	Variable	Study registry	Cost manual	€25.00
Work absenteeism males ≥ 35 years	Hours	Variable	Study registry	Cost manual	€39.00
Work absenteeism females < 35 years	Hours	Variable	Study registry	Cost manual	€24.00
Work absenteeism females ≥ 35 years	Hours	Variable	Study registry	Cost manual	€30.00

Number of units for fixed costs are displayed for early mobilization (EM) and plaster immobilization (PI)

^a^Protocolled radiographs prior to and after reduction and after 1 week

^b^Due to the low costs of medication an estimated average of €2 was maintained per case of anesthetics/sedation

^c^General hospital

^d^Academic hospital

^e^Protocol cost, only materials (OR use, anesthesiologist and surgeon wages not included) average time for plexus block 15 min. Average operating time for arthrolysis, ulnar nerve release and arthroscopy of the wrist were 240, 190 and 75 min, respectively

^f^Protocolled visits at 1 and 3 weeks

^g^Protocolled visits at 6 weeks, 3 months, 6 months and 1 year

^h^Protocolled radiographs at 1 year

^i^Visits as a result of adverse events

^j^Diagnostics as a result of adverse events

The costs for use of the operating room included cost for personnel, anesthesia (not including the wage of the anesthesiologist), and overhead costs. An estimation of these costs was made by calculating the means of the fixed cost prices, which were derived from four participating hospitals (one academic and three regional hospitals). Cost prices for other health care resources were derived from the Dutch manual on cost research [18]. Unit costs for all diagnostic procedures were derived from the Dutch Health Care Authority (NZa, Nederlandse Zorgautoriteit). Medication costs were calculated using standard unit prices as described by the CVZ (College voor zorgverzekeringen, Health Care Insurance Board; online available at https://www.medicijnkosten.nl). Indirect costs due to productivity loss were calculated using the friction cost method, which assumes that initial production levels restore after some period of adaption, taking economic circumstances into account [19].

### Statistical analysis

Analyses were performed using the Statistical Package for the Social Sciences (SPSS) version 21 (IBM Corp. Released 2011. IBM SPSS Statistics for Windows, Armonk, NY, USA). The FuncSiE trial was designed to enroll 100 patients. The sample size calculation was performed from a clinical perspective, and is published elsewhere [12, 13].

Analysis was by intention to treat and all statistical tests were two-sided. Missing data were not imputed. Chi-squared analysis was used for statistical testing of categorical data. Univariate analysis of continuous data was done using a Mann–Whitney *U* test (non-parametric data) or a Student’s *T* test (parametric data). *p* values < 0.05 were regarded as statistically significant. Accelerated bootstrapping was used for pairwise comparison of the mean differences in all hospital costs, out of hospital costs, indirect costs and total costs between the two treatment groups. The number of replications was chosen to be 1000.

SF-36 and EQ-5D were repeatedly measured over time, and were compared between treatment groups using linear mixed-effects regression models. These multilevel models included random effects for the intercepts of the regression model and time coefficient of individual patients. Since the outcome measures were not linearly related with time, the time points were entered as factor. The models included fixed effects for treatment group, involvement of the dominant side, and gender. The effect of age was non-significant in all models and age was therefore not included. The interaction between treatment group and time was included in the model to test for differences between the groups over time (i.e., differences in recovery time). For each follow-up moment, the estimated marginal mean of the EQ-5D utility score and the SF-36 physical component summery (PCS) and mental component summery (MCS) scores were computed per treatment group and compared post hoc using a Bonferroni test to correct for multiple testing. Absence of overlap in the 95% confidence interval around the marginal means was regarded as significant at *p* < 0.05.

## Results

**Fig. 1:**
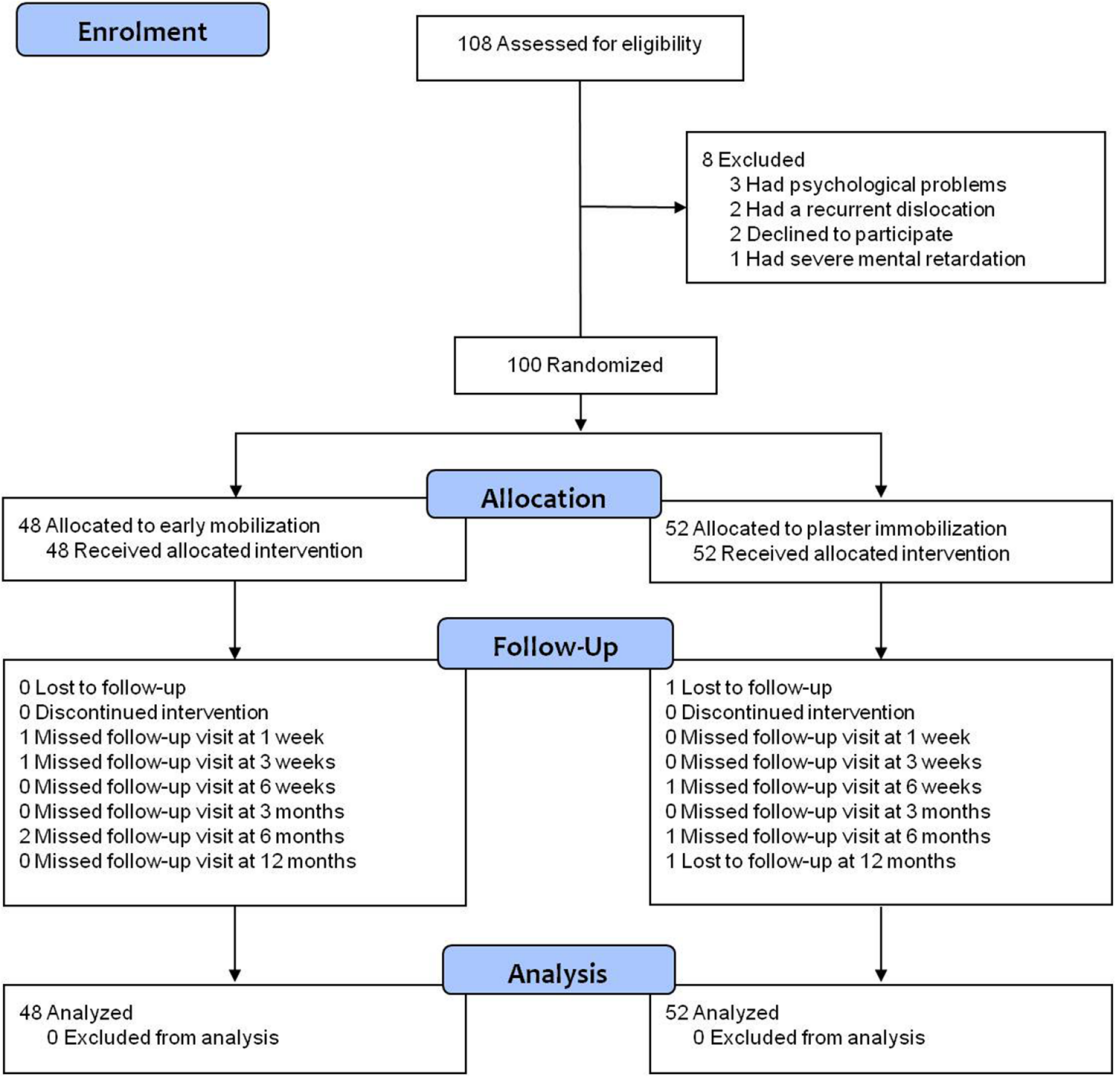
Trial flow chart

Of the hundred patients enrolled, 48 were assigned to early mobilization and 52 to plaster immobilization (Fig. 1). All patients received the allocated treatment. At 1 year follow-up, complete cost data were available for 99 patients; one patient in the plaster group was lost to follow-up after 6 months. Apart from a relative predominance of patients with an affected dominant side in the early mobilization group, randomization resulted in similar baseline and injury characteristics in the two groups (Table 2).

**Table 2:** Characteristics of trial participants by treatment group

	Early mobilization *N* = 48	Plaster immobilization *N* = 52
Patient characteristics		
Male^a^	22 (46%)	20 (39%)
Age^b^ (year)	43 (16)	47 (14)
Independent living^a^	44 (92%)	50 (96%)
Household composition^a^		
Alone	10 (21%)	10 (19%)
Alone with children	1 (2%)	3 (6%)
With partner	18 (38%)	19 (37%)
With partner and children	13 (27%)	17 (33%
With family/friends	6 (13%)	3 (6%)
Activities of daily living		
Work participation (*N* patients)^a^	32 (67%)	32 (62%)
Work participation (h/week)^c^	36.0 (24.0–40.0)	36.0 (24.0–40.0)
Injury characteristics		
Dominant side affected^a^	24 (50%)	22 (42%)
Reduction in operating room^a^	5 (10%)	1 (2%)
Reduction anesthesia^a^		
IV valium	21 (44%)	17 (33%)
General anesthesia	10 (21%)	8 (15%)
Intra-articular	3 (6%)	12 (23%)
None	6 (13%)	9 (17%)
Other	6 (13%)	6 (12%)
Regional/plexus	2 (4%)	0 (0%)

Data are presented as ^a^*N* (%), ^b^mean (SD), or ^c^median (*P*_25_–*P*_75_)

### Quality of life

No statistically significant differences in health-related quality of life measured with the EQ-5D and SF-36 between the two groups were noted throughout the 1-year follow-up (Table 3). The EQ-5D was consistently between 0.82 and 0.89 during follow-up. The SF-36 PCS varied between 42 and 53 and the SF-36 MCS varied between 55 and 59 throughout the whole follow-up. Both component summary scores remained within the population norm of 50 ± 10 (SD) points, and were independent of treatment.

**Table 3:** Health-related quality of life at all follow-up moments by treatment group

Outcome score	Follow-up	Early mobilization *N* = 48	Plaster immobilization *N* = 52
EQ-5D utility score	6 weeks	0.86 (0.83–0.89)	0.82 (0.79–0.85)
	3 months	0.87 (0.84–0.90)	0.86 (0.84–0.89)
	6 months	0.88 (0.86–0.91)	0.88 (0.85–0.91)
	12 months	0.88 (0.85–0.91)	0.89 (0.87–0.92)
SF-36 PCS	6 weeks	45 (43–48)	42 (40–44)
	3 months	52 (50–54)	50 (48–52)
	6 months	53 (50–55)	52 (50–54)
	12 months	53 (51–55)	53 (51–55)
SF-36 MCS	6 weeks	56 (54–58)	59 (57–61)
	3 months	57 (55–59)	57 (55–59)
	6 months	57 (55–59)	56 (54–58)
	12 months	55 (53–57)	56 (54–58)

Data are shown as the estimated marginal mean with 95% confidence interval adjusted for involvement of the dominant side and gender. None of the intervals overlapped indicating no statistical significant difference between the treatment groups

EQ-5D, EuroQoL 5D; SF-36, Short Form-36; PCS, Physical Component Summary score; MCS, Mental Component Summary score

### Health care costs

**Fig. 2:**
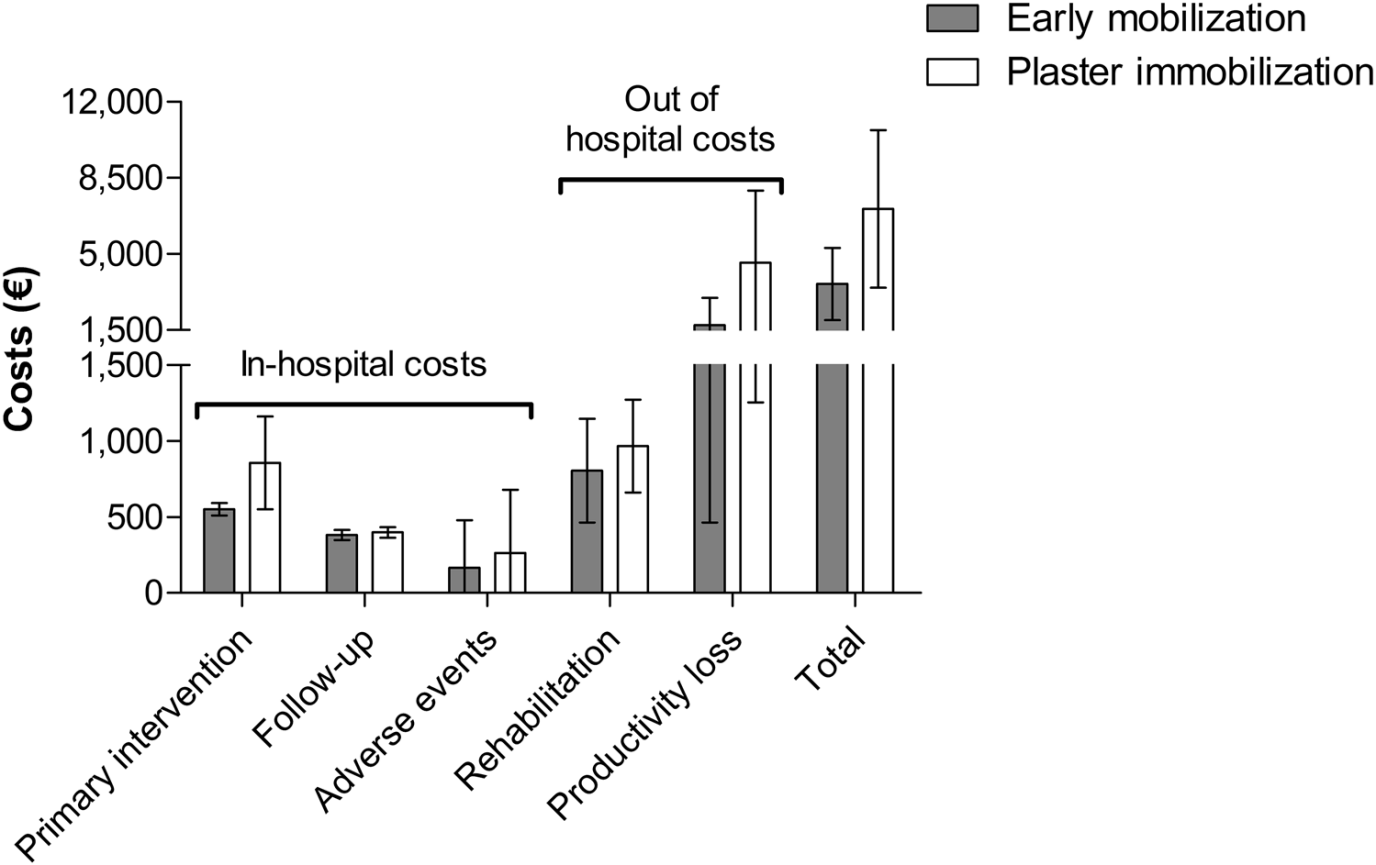
Mean total costs and costs per cost category by treatment group

Total costs and costs per category are shown in Table 4 and Fig. 2. The mean total costs per patient were €3624 (95% confidence interval (CI) 1966–5281) in the early mobilization group versus €7072 (95% CI 3444–10,701) in the plaster group. Although early mobilization was €3449 less expensive than plaster immobilization, this difference was not statistically significant (*p* = 0.094).

**Table 4:** Total costs and costs per cost category by treatment group

Cost categories	Early mobilization *N* = 48	Plaster immobilization *N* = 52	Difference	*p* value
Direct costs	€1904 (1303 to 2505)	€2483 (1822 to 3144)	− €579	0.198
Intramural costs	€1098 (785 to 1411)	€1517 (1004 to 2031)	− €419	0.173
Primary intervention	€551 (510 to 591)	€856 (551 to 1161)	− €305	0.058
Follow-up	€382 (349 to 415)	€399 (364 to 434)	− €17	0.481
Adverse events/revision surgery	€166 (− 147 to 478)	€263 (− 153 to 678)	− €97	0.712
Out of hospital costs (FU/rehabilitation)	€806 (465 to 1147)	€966 (660 to 1271)	− €160	0.483
Indirect costs (productivity loss)	€1719 (465 to 2974)	€4589 (1258 to 7920)	− €2870	0.120
Total	€3624 (1966 to 5281)	€7072 (3444 to 10,701)	− €3449	0.094

Data are shown as the mean costs per patient with 95% confidence interval given between brackets and were analyzed with a regression analysis after bootstrapping

FU, follow-up

The costs for the primary intervention were €551 (95% CI 510–591) in the early mobilization group versus €856 (95% CI 551–1161) in the plaster immobilization group (*p* = 0.058). Due to the identical, protocolled follow-up, there was no difference in the follow-up costs; €382 (95% CI 349–415) in the early mobilization group versus €399 (95% CI 364–434) in the plaster group (*p* = 0.481). Details concerning adverse events are shown in Table 5. Adverse events occurred in five patients in the early mobilization group versus seven patients in the plaster immobilization group. Costs for diagnosis and treatment of adverse events were €166 (95% CI − 147 to 478) in the early mobilization group versus €263 (95% CI − 153 to 678) in the plaster immobilization group (*p* = 0.712). The main determinant in the costs for adverse events were costs for surgery. This applied to three patients, one in the early mobilization group (€4744) versus two in the plaster immobilization group (€3007 and €1687).

**Table 5:** Adverse events and secondary interventions by treatment group

	Early mobilization *N* = 48	Plaster immobilization *N* = 52	*p* value
Adverse events	5 (10%)	7 (13%)	0.640
Secondary interventions (*N* patients)	1 (2%)	2 (4%)	1.000
Secondary interventions (*N* interventions)	1 (2%)	2 (4%)	1.000
Arthrolysis	1	0	
Ulnar nerve release	0	1	
Arthroscopy of the wrist	0	1	

Data are presented as *N* (%) and were analyzed using a Chi-squared test

The out of hospital costs during follow-up and rehabilitation were €806 (95% CI 465–1147) in the early mobilization group versus €966 (95% CI 660–1271) in the plaster group (*p* = 0.483). These costs were mainly due to physical therapy (€738 versus €808; *p* = 0.693; data not shown). This can be explained by the fact that most patients in both groups attended physical therapy to some degree.

### Productivity loss

Work absenteeism did not differ significantly between both groups, although the early mobilization group reported slightly less absenteeism (69% versus 78%; *p* = 0.572; Table 6). Patients who were treated with early mobilization resumed work 8 days sooner than did patients that were treated with plaster immobilization (10 versus 18 days; *p* = 0.027). The associated mean costs for lost productivity in the total study population were €1719 (95% CI 465–2974) in the early mobilization group versus €4589 (95% CI 1258–7920) in the plaster group. Despite the large difference of €2870 in favor of early mobilization, this did not reach statistical significance (*p* = 0.120). When considering only patients that reported sick, the mean costs for productivity loss per absentee were €3751 (95% CI 1174–6329) in the early mobilization group and €9546 (95% CI 2955–16,137) in the plaster group (*p* = 0.115).

**Table 6:** Work participation and resumption by treatment group

	Early mobilization *N* = 48	Plaster immobilization *N* = 52	*p* value
Work absenteeism (*N* patients)^A^	22 (69%)	25 (78%)	0.572^a^
Resumption at 12 months (*N* patients)^A^			
No	0 (0%)	1 (4%)	0.637^a^
Partial	1 (4%)	1 (4%)	
Fully	21 (96%)	23 (92%)	
Time of full resumption (days)^B^	10 (5–16)	18 (8–41)	0.027^b^
Hours resumed at 12 months (% of baseline)^B^	100 (100–100)	100 (100–100)	0.376^b^

Data are presented as ^A^*N* (%) or as ^B^median (*P*_25_–*P*_75_) and were analyzed using a ^a^Chi-squared test and ^b^Mann–Whitney *U* test, respectively

## Discussion

The FuncSiE trial already showed that patients following a simple elbow dislocation demonstrate earlier recovery of elbow function when treated with early mobilization compared with plaster immobilization. As a consequence, early mobilized patients were able to resume work 8 days earlier. Current data demonstrated that health-related quality of life at 1 year was similar in both groups. Early mobilization showed a consistent trend towards being a less expensive treatment than plaster immobilization for all cost categories studied, yet the difference did not reach statistical significance. Surprisingly, there was also no statistically significant difference in costs for physical therapy between the two groups, despite earlier recovery of elbow function in patients that were treated with early mobilization. This could be explained by the fact that both groups received physical therapy according to an identical treatment protocol. Therefore, these data do not allow to reliably answer the questions whether earlier functional recovery after early mobilization consequently leads to less physical therapy in terms of frequency and duration and whether in that way a reduction in care costs might be realized.

### Comparison with other studies

The only recent study on this subject is a retrospective study, which reported the direct healthcare costs of simple elbow dislocations [20]. They divided all patients into three groups according to duration of plaster immobilization (*I*: < 2 weeks, *n* = 26, II: 2–3 weeks; *n* = 27 and III: > 3 weeks; *n* = 14). They concluded that the length of elbow immobilization did not influence the medical costs. The median direct costs for a simple elbow dislocation in their population were €1375, whereas this was €1904 and €2483 (early mobilization and plaster, respectively) in our study population. Their retrospective study design could explain this difference, as certain cost categories are almost impossible to collect from hospital charts only, which inevitably introduced bias. Another explanation for higher direct costs in our study could be that patients in our study had a protocolled follow-up period of 1 year. This implied that patients visited the out-patient clinic even when the elbow was considered as completely recovered, leading to costs that would otherwise not have been made. No studies report the indirect costs due to productivity loss as a result of simple elbow dislocations.

### Strengths and limitations

The most important drawback of this study concerns the absence of significance in the substantial differences between costs in both groups. This was mainly caused by the fact that only three patients underwent surgery as a result of adverse events. This led to excessive total costs for these patients compared with patients who healed uneventfully. These outliers caused considerable variation in costs which could falsely have led to the conclusion that the study lacked power. Moreover, the sample size calculation of the trial was performed from a clinical perspective rather than for cost calculation purposes. Statistically significant difference in total costs could have been demonstrated provided each treatment group should have encompassed 134 patients (*β* = 0.8, *α* = 0.05 and two-sided testing). A larger sample size would possibly have captured additional complications, altering direct and indirect costs associated with the care of simple elbow dislocations.

Unfortunately, these data did not allow to calculate the cost-effectiveness and cost–utility ratio of early mobilization, as there was no statistically significant difference in health-related quality of life at 1-year follow-up between both groups. On the other hand, there is no relevance in performing a cost-effectiveness or cost–utility analysis for a treatment that leads to earlier functional recovery at almost half the costs per patient (€3449 less expensive).

A strength of this study is the data completeness. All data were prospectively collected during the entire rehabilitation process, thus giving a truthful reflection of the actual total costs following a simple elbow dislocation. The incidence rate of elbow dislocations in the Netherlands is 5.6 (per 100,000 person years) [2]. The difference of €3449 in total costs was not statistically significant, but changing treatment protocols for simple elbow dislocations could, in the current Dutch population (16.8 million persons, source: https://www.cbs.nl/nl-NL/menu/cijfers/default.htm, last update April 2014), reduce the care costs by at least 3.2 million euro per year, supporting the societal relevance of early mobilization.

## Conclusion

The results of this study show that early mobilization of adult patients with a simple elbow dislocation leads to earlier resumption of activities of daily living and work, which might reduce costs by approximately 50%. The results of the FuncSiE trial provided clinical evidence supporting early mobilization after simple elbow dislocations. Current analysis proved that early mobilization should also be the treatment of choice for this injury from a socio-economic point of view.
